# Infections in Chronic Lymphocytic Leukemia: Evolving Risks and Prevention Strategies

**DOI:** 10.1111/ejh.70065

**Published:** 2025-11-25

**Authors:** Enrica Antonia Martino, Santino Caserta, Ernesto Vigna, Antonella Bruzzese, Nicola Amodio, Eugenio Lucia, Virginia Olivito, Caterina Labanca, Francesco Mendicino, Fortunato Morabito, Massimo Gentile

**Affiliations:** ^1^ Hematology Unit, Department of Onco‐Hematology AO of Cosenza Cosenza Italy; ^2^ Department of Experimental and Clinical Medicine University of Catanzaro Catanzaro Italy; ^3^ AIL Sezione di Cosenza Cosenza Italy; ^4^ Department of Pharmacy, Health and Nutritional Science University of Calabria Rende Italy

**Keywords:** Bruton's tyrosine kinase inhibitors, chronic lymphocytic leukemia, immunodeficiency, infection prophylaxis, vaccination

## Abstract

Infections remain a leading cause of morbidity and mortality in patients with chronic lymphocytic leukemia (CLL), reflecting both intrinsic immune dysfunction and therapy‐related immunosuppression. The pathogenesis of immunodeficiency in CLL is multifactorial: neoplastic B cells impair humoral immunity, T cells are functionally exhausted, and innate immune cells, particularly neutrophils and NK cells, display profound defects. Beyond impaired pathogen defense, these immune alterations actively support leukemic cell survival and promote a tolerogenic microenvironment. The advent of targeted therapies has reshaped the infectious risk profile. Bruton's tyrosine kinase inhibitors (BTKis) and venetoclax have largely replaced chemotherapy, reducing classic opportunistic infections but introducing new challenges. BTKis are associated with invasive fungal infections and increased pneumonia risk in combination regimens, while venetoclax frequently induces profound neutropenia. Anti‐CD20 monoclonal antibodies cause long‐lasting B‐cell depletion and viral reactivation. These evolving risks demand nuanced approaches to prevention. Prophylactic strategies must be individualized. Antiviral prophylaxis is warranted with anti‐CD20 antibodies and BTKis, and Pneumocystis jirovecii pneumonia (PJP) prophylaxis remains essential with fludarabine, cyclophosphamide, and rituximab (FCR) or prolonged corticosteroid therapy. Antifungal prophylaxis is not routinely indicated in CLL but may be considered in high‐risk patients on BTKis or with refractory disease. Immunoglobulin replacement therapy (IgRT) reduces recurrent bacterial infections in patients with hypogammaglobulinemia, while vaccination—though often limited by suboptimal responses—remains the cornerstone of prevention. Timing before therapy or during treatment‐free intervals is critical, and newer formulations, such as conjugate pneumococcal and recombinant zoster vaccines, are preferred. Future directions include developing predictive biomarkers of infection risk and vaccine responsiveness, integrating immune monitoring into clinical trials, and exploring strategies to modulate neutrophil plasticity and restore T‐cell function. Until then, a pragmatic, risk‐adapted approach combining vigilance, prophylaxis, immunoglobulin replacement, and optimized vaccination offers the best safeguard for patients with CLL.

## 1 Introduction

Chronic lymphocytic leukemia (CLL) is among the most common mature lymphoid malignancies in adults in Western countries [1] and represents a paradigmatic example of cancer‐associated immune dysfunction characterized by profound alterations in both innate and adaptive immunity [2]. Despite the remarkable progress in the treatment of CLL over recent decades, infections persist as the leading cause of mortality in this patient population [3, 4]. The intrinsic linkage to immune dysfunction manifests early in the disease and progressively worsens over time. Even in the absence of treatment, patients exhibit immunologic deficiencies that compromise host defense, increasing the risk of infections, impairing vaccine responses, and contributing to secondary malignancies [5]. The immune dysfunction observed in CLL is complex and multifactorial, arising from both the intrinsic immune defects of the disease and the possible immunosuppressive effects of therapy [6, 7]. These include hypogammaglobulinemia, impaired cell‐mediated immunity, and complement system abnormalities, all of which interact with the tumor microenvironment (TME) to further compromise host defenses [6, 7, 8, 9]. Patients with CLL are at increased risk for infections at all stages of the disease, and both the disease and its treatment contribute to this vulnerability [10, 11]. Notably, hypogammaglobulinemia is associated with worse survival outcomes [12]. Epidemiological data suggest that immune dysfunction may even precede the clinical diagnosis of CLL [13, 14]. Innate immune defects include diminished complement activity—both quantitative and functional—as well as impaired neutrophil and monocyte function. This leads to poor opsonization and reduced microbial clearance. In parallel, adaptive immunity is compromised by defective T‐cell function and hypogammaglobulinemia, which are hallmarks of advanced CLL. CD4+ T‐cell depletion, expansion of exhausted CD8+ subsets, and dysregulation of cytokine signaling contribute to impaired immune surveillance [13, 14].

Therapeutic intervention often amplifies this preexisting immune suppression. Chemoimmunotherapy regimens historically used in CLL, such as fludarabine, cyclophosphamide, and rituximab (FCR), or bendamustine with rituximab (BR), profoundly affect both B and T cell compartments. Fludarabine is known to cause deep and prolonged T‐cell lymphopenia, while rituximab depletes normal B cells, exacerbating hypogammaglobulinemia and impairing humoral immunity. Bendamustine further contributes to immunosuppression through its cytotoxic effects on lymphocytes, particularly CD4+ T cells, and is frequently associated with long‐term lymphopenia [15].

A study by Svanberg Teglgaard R, et al. showed that, beyond their direct anti‐leukemic effects, new drugs can reverse disease‐associated innate immune dysfunction [16]. The demonstration that both BTK inhibitor monotherapy and BTK/BCL2 inhibitor combination therapy rapidly normalize impaired cytokine responses and myeloid cell activation is particularly noteworthy, as infections remain a leading cause of morbidity and mortality in CLL. The use of real‐time, clinically implementable immune assays strengthens the translational relevance of these findings. Importantly, the correlation between improved innate immunity and reduced infection burden suggests that targeted agents may offer dual benefits in CLL: disease control and immune restoration. This has practical implications for patient management, especially for those with a history of recurrent infections or high infectious risk. The study also raises the possibility that short‐term targeted therapy could be considered in selected patients to mitigate infection risk, a hypothesis currently being tested in the PreVent‐ACaLL trial [17]. While the sample size is modest and longer‐term follow‐up is needed, these results suggest that immune monitoring may represent a useful component of CLL care, particularly for patients at increased risk of infections.

Together, these findings underscore that immune dysfunction in CLL is not solely a consequence of the leukemic clone but is dynamically shaped by therapeutic intervention. Recognizing the immunologic sequelae of both conventional and targeted therapies is essential for optimizing infection prevention strategies and for supporting immune recovery across the disease continuum.

In parallel, prophylactic measures and vaccination strategies have become more nuanced. As traditional chemotherapy regimens wane in popularity, attention has shifted toward redefining prophylaxis and immunization in the context of oral targeted agents and prolonged treatment durations. Moreover, the COVID‐19 pandemic has heightened awareness of the need to optimize vaccine responses in immunocompromised individuals, such as those with CLL [18, 19, 20, 21, 22, 23, 24, 25, 26, 27]. This review aims to provide an updated overview of infection pathogenesis in CLL, evaluate the current evidence guiding prophylactic interventions, and critically examine the role of vaccination in this vulnerable population.

## 2 Mechanisms of Immunodeficiency in CLL

Immune impairment in CLL is multifactorial, affecting both the innate and adaptive branches of the immune system [28]. CLL cells disrupt immune homeostasis through direct cellular interactions and secretion of immunomodulatory cytokines. Defects in innate immunity—such as impaired natural killer (NK) cell function, neutrophil and monocyte abnormalities, and complement deficiencies—reduce the host's ability to mount an effective response to pathogens [29, 30, 31, 32, 33, 34]. Adaptive immunity is also compromised, with dysfunctional antigen presentation and antibody production. These abnormalities are present even in untreated patients, indicating that immune dysfunction is intrinsic to CLL and not merely a consequence of therapy. The TME further amplifies immune suppression, perpetuating a cycle of impaired pathogen defense and increased infection risk [35, 36, 37, 38].

The expansion of neoplastic B cells in CLL leads to the displacement of normal B lymphocytes, fundamentally altering humoral immunity [35, 39, 40]. CLL cells, driven by chronic B‐cell receptor (BCR) signaling and interactions within the TME, outcompete their normal counterparts and fail to generate a diverse and effective antibody repertoire. This monoclonal dominance impairs the production of pathogen‐specific antibodies and undermines the host's ability to respond to new antigens. The inability of CLL cells to support normal humoral immunity is a central factor in the heightened susceptibility to infections observed in this patient population [41, 42].

The TME in CLL is a complex ecosystem comprising CLL cells, T cells, nurse‐like cells, stromal elements, and a milieu of cytokines and chemokines [35, 36, 37, 38]. CLL cells secrete anti‐inflammatory cytokines such as IL‐10 and proinflammatory cytokines like IL‐6, fostering an immunosuppressive milieu [43, 44]. They also recruit and modulate bystander cells, including regulatory T cells (Tregs) and myeloid‐derived suppressor cells (MDSCs), which further inhibit effective immune responses [45, 46, 47, 48]. The TME orchestrates a state of immune tolerance, supporting CLL cell survival and proliferation while blunting the host's capacity to clear infections or mount anti‐tumor immunity [49, 50].

A hallmark of CLL‐associated immune dysfunction is hypogammaglobulinemia, particularly involving reductions in IgG and IgA [51, 52]. The Ig deficit is multifactorial, arising from both the loss of normal B cells and the suppressive effects of CLL cells on plasma cell differentiation and immunoglobulin production [8, 10]. Hypogammaglobulinemia is a strong predictor of infection risk, with lower immunoglobulin levels correlating with more frequent and severe infections, a need for earlier treatment [53], and reduced survival [8, 10, 52].

A real‐world study investigated the patterns and clinical impact of IgG testing and immunoglobulin replacement therapy (IgRT) in patients with CLL and non‐Hodgkin lymphoma [51]. In this retrospective cohort, only about two‐thirds underwent IgG testing at any point, and just 6.5% received IgRT, despite a third of those tested being found to have hypogammaglobulinemia. Notably, IgG testing was often delayed after diagnosis, and the number of tests per patient varied widely. Among those who did receive IgRT, the therapy was associated with a significant and sustained increase in IgG levels and a marked reduction in the prevalence of hypogammaglobulinemia at 3, 6, and 12 months post‐initiation. For example, the proportion of CLL patients with hypogammaglobulinemia dropped from 85% to 66% at 3 months, and similar improvements were seen at later time points. Crucially, IgRT was also associated with substantial reductions in both overall and severe infections, as well as a decrease in the use of antimicrobials for infection management. The odds of any infection or severe infection in CLL patients were halved after IgRT initiation, and these benefits were consistent across multiple time points. Furthermore, the study found that more frequent IgG testing was independently associated with a lower risk of severe infection, likely reflecting both better detection of hypogammaglobulinemia and more timely use of IgRT. Despite these benefits, only a minority of CLL patients with hypogammaglobulinemia and recurrent or severe infections actually received IgRT, suggesting a gap between guideline recommendations and real‐world practice. The authors highlight the need for more standardized protocols and greater awareness among clinicians to optimize the management of secondary immune deficiency in CLL.

Finally, complement deficiencies—especially within the classical pathway—diminish opsonization and hinder the clearance of encapsulated bacteria. This adds to the already elevated risk of pneumonia and other invasive bacterial infections, a leading cause of hospitalization and mortality in CLL patients [54]. Table 1 illustrates the disrupted multiple components of the immune system, leading to an increased susceptibility to infections and suboptimal responses to immunization.

**TABLE 1:** Immune defects in chronic lymphocytic leukemia and their clinical consequences.

Immune component	Defect	Clinical consequence
B cells	Hypogammaglobulinemia	Recurrent bacterial infections, poor vaccine responses
T cells	CD4/CD8 imbalance, functional exhaustion	Increased viral infections (HSV, CMV, VZV)
Neutrophils	Reduced chemotaxis, oxidative burst	Poor bacterial clearance, fungal infections
NK cells	Impaired cytotoxicity	Viral reactivation (E.G., EBV, CMV)
Complement	Classical pathway deficiency	Susceptibility to encapsulated organisms

## 3 Dual Faces of Neutrophils in CLL: Immune Dysfunction and Tumor Support

Traditionally, the focus has been on the adaptive immune compartment—impaired B‐cell antibody production, exhausted T cells, and dysfunctional NK cells. However, this recent review draws attention to neutrophils, the most abundant innate immune cells, and reveals how their biology is deeply perturbed in CLL.

Neutrophils, ordinarily regarded as first‐line defenders against infection, display a paradoxical duality in CLL [55]. On the one hand, their classical functions—including phagocytosis, chemotaxis, and reactive oxygen species production—are impaired, leaving patients vulnerable to recurrent and severe infections. On the other hand, these same neutrophils are “reprogrammed” by the leukemic microenvironment into cells that paradoxically support tumor progression. Increased formation of neutrophil extracellular traps (NETs), for example, not only fails to clear pathogens effectively but also promotes leukemic B‐cell survival and contributes to the immunosuppressive milieu.

McManus et al. [55] carefully detail the molecular and cellular cross‐talk that underpins this transformation. CLL B cells secrete cytokines such as IL‐10, G‐CSF, and GM‐CSF that prolong neutrophil survival and induce the emergence of immunosuppressive subsets, such as CD16^high^CD62L^dim^ neutrophils. In return, these neutrophils enhance leukemic cell fitness by delivering survival signals through BAFF, APRIL, and downstream NF‐κB and PI3K/AKT/mTOR pathways. Moreover, neutrophils exert inhibitory effects on T cells, upregulating checkpoint molecules like PD‐L1 and releasing NETs that can directly induce T‐cell apoptosis. Thus, rather than acting solely as defenders against microbial invaders, neutrophils in CLL are woven into the fabric of tumor‐supportive immune dysfunction.

Therapy further complicates this picture. Classical chemotherapy, such as fludarabine or alkylating agents, causes neutropenia and predisposes patients to bacterial and opportunistic infections. Anti‐CD20 monoclonal antibodies bring risks of late‐onset neutropenia and viral reactivation. Modern targeted therapies, though transformative in efficacy, are not exempt from immunological costs: Bruton's tyrosine kinase inhibitors such as ibrutinib compromise neutrophil functions, including ROS generation and phagocytosis, explaining the surge in invasive fungal infections observed in treated patients. PI3K inhibitors similarly heighten vulnerability to viral and fungal pathogens. Thus, CLL patients face a double jeopardy: neutrophils rendered ineffective by the disease itself and further impaired by life‐prolonging therapies.

Against this backdrop, McManus also highlights promising avenues for therapeutic innovation. By targeting neutrophil biology, and particularly targeting aberrant NET formation, it emerges as a tantalizing prospect [55]. PAD4 inhibitors, nanoparticle‐mediated delivery of sivelestat, and repurposing drugs such as disulfiram are under investigation to curb excessive NETosis. At the same time, efforts to manipulate neutrophil polarization—shifting tumor‐promoting subsets into anti‐tumor phenotypes—could open a novel immunotherapeutic strategy for CLL.

From an expert perspective, what stands out most is the need to reframe how we view neutrophils in CLL. They should no longer be regarded as passive victims of treatment‐related cytopenia but as active participants in the disease process. Their contribution to both infection risk and leukemic progression places them at the crossroads of immunity and malignancy. This recognition has important clinical implications: neutrophil‐related biomarkers could enrich prognostic models; infection prophylaxis strategies may need to be tailored not only to drug‐induced cytopenia but also to functional defects; and therapies aimed at restoring neutrophil competence could synergize with BCR‐targeted agents to improve disease control.

Ultimately, this review makes a persuasive case that neutrophils are key architects of the immune landscape in CLL. Their dysfunction, both intrinsic and therapy‐induced, is a major driver of morbidity and a potential contributor to disease persistence. Understanding and targeting these cells may therefore represent the next frontier in refining treatment strategies, reducing infection‐related complications, and perhaps even dismantling the supportive microenvironment that allows CLL to thrive.

## 4 Infection Risk During the CLL Disease Course

The infectious burden in CLL is dynamic and highly dependent on treatment history. In early‐stage, treatment‐naïve patients, the most frequent infections are community‐acquired respiratory tract infections, often caused by *Streptococcus pneumoniae* or *Haemophilus influenzae* . These are largely driven by hypogammaglobulinemia and T‐cell impairment [7].

In contrast, patients undergoing chemotherapy—especially fludarabine‐based regimens such as FCR [56]—face a markedly higher risk for opportunistic infections, including Pneumocystis jirovecii pneumonia (PJP), CMV reactivation, and invasive fungal infections. These regimens result in prolonged CD4+ lymphopenia, neutropenia, and further depression of B‐cell immunity.

Targeted therapies have altered the infection landscape. BTK inhibitors such as ibrutinib and acalabrutinib are associated with a modestly increased risk of invasive fungal infections, notably aspergillosis, especially early in therapy or in heavily pretreated patients [57].

Venetoclax, particularly in combination with anti‐CD20 monoclonal antibodies, can lead to profound neutropenia, necessitating vigilance for bacterial and fungal infections [58].

Anti‐CD20 monoclonal antibodies, including rituximab and obinutuzumab, are potent B‐cell depleting agents and contribute to long‐lasting impairment in humoral immunity. One major risk associated with their use is hepatitis B virus (HBV) reactivation, which may lead to fulminant hepatitis and death if not prevented [59]. Table 2 summarizes the results.

**TABLE 2:** Infection risk profile by treatment type in CLL.

Treatment modality	Common infections	Comments
Watch & Wait	Respiratory infections	Mostly bacterial, linked to Hypogammaglobulinemia
FCR	PJP, CMV, Bacterial, Fungal	Profound Lymphopenia, neutropenia
BTK inhibitors	Aspergillosis, Pneumonia	Early risk, especially in prior‐treated patients
Venetoclax ± anti‐CD20	Bacterial, Fungal	Risk during neutropenic periods
Anti‐CD20 antibodies	HBV reactivation, VZV	Long‐lasting B‐cell depletion

## 5 Infectious Risk Profile of BTK Inhibitors

The introduction of BTKis has transformed the management of CLL, offering highly effective, chemotherapy‐free alternatives across both frontline and relapsed/refractory settings. As these agents have moved to the forefront of clinical practice, concerns have emerged regarding their infectious toxicity profile—particularly in light of the baseline immune dysfunction inherent to CLL/SLL and the immunomodulatory effects of BTK inhibition.

To systematically assess these risks, Buchrits et al. conducted a comprehensive meta‐analysis of 18 randomized controlled trials (RCTs), encompassing 8324 patients [48, 60] Table 3 summarizes the results of this meta‐analysis. The included studies compared BTKi‐containing regimens—administered as monotherapy or in combination with anti‐CD20 antibodies or venetoclax—to various comparators, including chemotherapy, chemoimmunotherapy, or other targeted agents. The analysis also incorporated head‐to‐head comparisons among different BTKis and between monotherapy and combination approaches. The meta‐analysis evaluated a broad spectrum of infection‐related outcomes. BTKi monotherapy was not associated with a statistically significant increase in the risk of any infection (RR 1.12, 95% CI: 0.94–1.34) or grade 3–4 infections (RR 1.05, 95% CI: 0.76–1.44) when compared with chemotherapy‐based or other regimens. Likewise, BTKi combinations with anti‐CD20 antibodies or venetoclax did not significantly elevate infection risk (RR 0.93, 95% CI: 0.79–1.09). Rates of sepsis and infection‐related mortality were also comparable across treatment arms.

**TABLE 3:** Infectious risks with BTKi‐containing regimens in CLL/SLL.

Comparison group	Any infection (RR, 95% CI)	Grade 3–4 infection (RR, 95% CI)	Pneumonia (RR, 95% CI)	Sepsis (RR, 95% CI)	Febrile neutropenia (RR, 95% CI)	Fatal infection (RR, 95% CI)	Key findings/Notes
BTKi monotherapy vs. other regimens	1.12 (0.94–1.34)	1.05 (0.76–1.44)	Not significant	0.50 (0.25–1.01)	0.32 (0.18–0.59)	Not significant	No increased risk of infection or severe infection; reduced febrile neutropenia
BTKi + anti‐CD20 vs. other regimens	0.93 (0.79–1.09)	NS	2.18 (1.29–3.70)	0.48 (0.12–1.84)	0.41 (0.29–0.59)	Not significant	Increased pneumonia risk; less febrile neutropenia
BTKi + venetoclax vs. other regimens	NS	NS	Not significant	Not significant	0.18 (0.08–0.40)	Not significant	Less febrile neutropenia
BTKi + anti‐CD20 + venetoclax vs. others	NS	NS	Not significant	Not significant	Not significant	Not significant	Insufficient data
Ibrutinib vs. other BTKis	NS	Not significant	Not significant	Not significant	Not significant	Not significant	Insufficient data
BTKi vs. BTKi + anti‐CD20	NS	1.02 (0.66–1.57)	Not significant	2.24 (0.99–5.08)	Not significant	Not significant	Possible trend toward more sepsis with the combination
BTKi + chemotherapy vs. chemotherapy	NS	Not significant	Not significant	Not significant	Not significant	Not significant	No difference in overall or severe infection risk

Abbreviations: CI, Confidence Interval; NS, no statistically significant difference or insufficient data; RR, Risk Ratio.

A key exception emerged concerning pneumonia, which was significantly more common in patients receiving BTKi plus anti‐CD20 combinations (RR 2.18, 95% CI: 1.29–3.70). This clinically relevant finding warrants increased surveillance for respiratory complications, particularly in patients with existing pulmonary comorbidities or prior therapy. The mechanism behind this elevated pneumonia risk remains unclear but may involve additive immunosuppressive effects or drug‐specific pulmonary toxicity, as previously observed with agents like ibrutinib.

In contrast, febrile neutropenia was significantly less frequent in patients treated with BTKi‐containing regimens compared to those receiving conventional chemotherapy or chemoimmunotherapy. This advantage is particularly important in older adults or those with comorbidities, for whom myelosuppression poses substantial clinical risk.

This meta‐analysis represents a valuable contribution to the evolving understanding of infectious complications associated with targeted therapies. Its strengths include the inclusion of a large number of contemporary RCTs and the robust methodology employed. However, limitations remain—notably the heterogeneity among included studies, the predominance of chemotherapy comparators, and the limited availability of pathogen‐specific data. These factors restrict the ability to precisely define the infectious spectrum associated with BTKi use.

As the field moves toward fixed‐duration therapy and more sophisticated combination strategies, future trials should prioritize direct comparisons between BTKis and other novel agents, such as venetoclax‐based regimens, with systematic reporting of infection‐related endpoints. More granular data on microbiological etiology, timing of infections, and predisposing risk factors will be crucial to refining prevention and management strategies.

In conclusion, this meta‐analysis provides reassuring evidence that BTKi‐based therapies—whether as monotherapy or in combination—do not confer an increased overall risk of infections or severe infections compared to traditional chemoimmunotherapy. Importantly, the increased risk of pneumonia in combination regimens highlights the need for careful patient selection and proactive monitoring. These findings, summarized in Table 3, support the continued use of BTKis as a safe and effective cornerstone of CLL/SLL treatment, while emphasizing the importance of ongoing vigilance and individualized infectious risk assessment.

## 6 Principles of Infectious Prophylaxis in CLL

A recent updated ECIL guideline provides a comprehensive review of primary antifungal prophylaxis in patients with hematological malignancies, reflecting the evolving landscape of both antineoplastic and antifungal therapies [61]. The recommendations are based on a rigorous literature review and consensus among European experts, with particular emphasis on new targeted therapies and cellular treatments. For CLL, the incidence of invasive fungal disease (IFD) remains relatively low (0.5%–3%) but varies according to treatment modality. Patients treated with BTKis, such as ibrutinib, exhibit a higher incidence of IFD (2%–3%) compared to those receiving conventional therapies. The guideline notes that antifungal prophylaxis is not routinely recommended for CLL patients (D‐III) but may be considered in selected high‐risk cases—such as those with refractory disease, prolonged neutropenia, or those receiving BTKis (C‐II). In cases where venetoclax is co‐administered, caution is advised due to significant drug–drug interactions, and dose adjustments guided by therapeutic drug monitoring are recommended.

Overall, this nuanced approach to antifungal prophylaxis in CLL is appropriate: while the overall risk of IFD is low, recognizing that BTKIs and prolonged neutropenia can elevate this risk is crucial for individualized patient management. The recommendation against routine prophylaxis in most CLL patients is evidence‐based and helps avoid unnecessary drug exposure, toxicity, and drug interactions. However, the guideline wisely allows for clinical judgment in high‐risk or refractory cases, especially as the population of heavily pretreated CLL patients continues to grow.

Antimicrobial prophylaxis is generally reserved for high‐risk patients, such as those with prolonged neutropenia or on specific therapies [62]. Importantly, targeted therapies may partially restore immune function by reducing tumor burden and normalizing immune cell populations, offering hope for improved infection outcomes in the future.

The cornerstone of infection prevention in CLL is risk stratification based on disease burden, treatment regimen, and prior infection history. Prophylaxis must be tailored accordingly.

Antibacterial prophylaxis is not routinely recommended for all patients but may be considered in those with recurrent bacterial infections or prolonged neutropenia. In such cases, fluoroquinolones or trimethoprim‐sulfamethoxazole (TMP‐SMX) may be employed, though antibiotic resistance remains a concern.

Antiviral prophylaxis with acyclovir or valacyclovir is generally recommended for patients receiving purine analogs, whereas its use in those treated with anti‐CD20 therapy, BTK inhibitors, or venetoclax should be individualized based on patient‐specific risk factors. In selected cases, this approach may help reduce the risk of herpes simplex virus (HSV) or varicella‐zoster virus (VZV) reactivation [63].

Antifungal prophylaxis is generally reserved for high‐risk patients, such as those with a history of invasive fungal infections or prolonged neutropenia. PJP prophylaxis with TMP‐SMX is standard in patients treated with FCR or receiving high‐dose corticosteroids. Alternatives such as atovaquone, dapsone or aerosolized pentamidine (300 mg monthly) may be considered in cases of sulfa allergy [64].

Agents such as posaconazole or fluconazole may be used, although data specific to CLL is limited.

Immunoglobulin replacement therapy is effective in reducing the frequency of bacterial infections in patients with documented hypogammaglobulinemia and recurrent infections. Both intravenous and subcutaneous formulations are used, with no clear superiority between the two [65].

Table 4 summarizes the main recommended prophylactic measures in CLL by treatment and risk category.

**TABLE 4:** Prophylactic measures in CLL by treatment and risk category.

Risk/treatment context	Prophylactic strategy	Agent/action
FCR, Corticosteroids	PJP Prophylaxis	TMP‐SMX, Alternative agents
ANTI‐CD20 therapies	Antiviral (HSV/VZV) + HBV Screening	Acyclovir, Lamivudine/Entecavir as needed
BTK/Venetoclax therapy	Antiviral, monitor fungal risk	Consider acyclovir, antifungals in high‐risk
Hypogammaglobulinemia + recurrent infections	Immunoglobulin replacement	IVIG or SCIG

## 7 Vaccination in CLL: A Critical Preventive Tool

Addressing immune dysfunction in CLL requires a multifaceted approach. Vaccination is recommended for all patients, though responses are often suboptimal due to underlying immune defects and the effects of therapy. Immunoglobulin replacement may benefit select patients with recurrent infections and hypogammaglobulinemia, but its impact on severe infections and overall survival is limited [64, 65]. Despite the known limitations in vaccine response among CLL patients, vaccination remains a central preventive measure. The timing of vaccination is paramount—ideally prior to treatment initiation or during treatment‐free intervals to maximize immunogenicity [66].

SARS‐CoV‐2 vaccines have received intense scrutiny in the CLL population. Multiple studies have shown that humoral response rates after mRNA vaccines are poor, particularly in patients on anti‐CD20 antibodies (≤ 10%) and BTK inhibitors (20%–40%) [67]. However, T‐cell responses may still develop and confer partial protection. Additional boosters and monoclonal antibody prophylaxis (e.g., tixagevimab/cilgavimab) are strategies to enhance protection in non‐responders [68].

Annual inactivated influenza vaccination is recommended, though seroconversion rates are modest (20%–40%). High‐dose or adjuvanted formulations may provide better protection [66].

Pneumococcal vaccination is essential, and conjugate vaccines such as PCV15 or PCV20 are preferred over polysaccharide vaccines. A sequential schedule—PCV followed by PPSV23—is recommended by most guidelines [69].

Herpes zoster vaccination with the recombinant subunit vaccine (Shingrix) is preferred and has demonstrated efficacy in immunocompromised hosts. It is indicated in patients over 50 years and those initiating therapies associated with zoster risk [70].

Hepatitis B vaccination should be offered to all seronegative patients prior to therapy, especially those planned for anti‐CD20 treatment. Double‐dose or accelerated schedules may improve seroconversion [71].

Other vaccines, including anti‐Tetanus, Diphtheria, Pertussis (Tdap) and anti‐ *Haemophilus influenzae* B (Hib), may be indicated depending on age, treatment status, and comorbidities.

Table 5 summarizes vaccine recommendations for CLL patients.

**TABLE 5:** Vaccine recommendations for CLL patients.

Vaccine	Indication	Timing	Notes
SARS‐COV‐2	All CLL patients	Before/during treatment	MRNA preferred, additional boosters needed
Influenza	All annually	Preferably before therapy	High‐dose or adjuvanted forms are better
Pneumococcal	All CLL patients	PCV15/20 then PPSV23	Repeat every 5–10 years as indicated
Herpes zoster (RZV)	> 50 years or planned treatment	Pre‐therapy	Recombinant only (SHINGRIX)
Hepatitis B	Seronegative prior to anti‐CD20	Before treatment	Accelerated/double‐dose schedule preferred

## 8 Conclusions and Future Directions

Infections remain a dominant cause of morbidity and mortality in CLL, reflecting both intrinsic immune dysfunction and therapy‐induced suppression. The treatment landscape has changed dramatically, with targeted therapies reducing some traditional risks but introducing new ones—such as invasive fungal infections with BTK inhibitors, neutropenia with venetoclax, and viral reactivation with anti‐CD20 antibodies. Neutrophils, once considered bystanders, are now recognized as active contributors to both impaired antimicrobial defense and tumor support, placing them at the crossroads of immunity and malignancy.

Prophylaxis has therefore become more complex. Updated guidelines recommend a selective approach: antifungal prophylaxis is not routine but may be considered in high‐risk patients on BTK inhibitors or with prolonged neutropenia; antiviral prophylaxis is indicated with anti‐CD20 antibodies and BTKis; and PJP prophylaxis remains essential with FCR or high‐dose corticosteroids. Immunoglobulin replacement remains effective in reducing bacterial infections in patients with hypogammaglobulinemia and recurrent infections, while vaccination—despite suboptimal responses—remains the cornerstone of prevention. Optimizing timing, favoring conjugate pneumococcal vaccines, recombinant zoster, hepatitis B, and SARS‐CoV‐2 vaccines, and exploring boosters or monoclonal antibody prophylaxis are key steps forward.

Looking ahead, three priorities stand out. First, personalized risk stratification, integrating immunoglobulin levels, immune cell function, and neutrophil competence, will allow tailored prevention. Second, standardized monitoring in clinical trials is needed to define therapy‐specific infectious risks more precisely. Third, novel immune‐restorative strategies—from enhancing vaccine responses and refining immunization schedules to modulating neutrophil and T‐cell dysfunction—represent a major frontier. Research into predictive biomarkers, microbiota–immune interactions, and vaccine responsiveness could help move the field toward individualized infection prevention.

Until such strategies are validated, a pragmatic, risk‐adapted approach—combining vigilance, prophylaxis, and vaccination—remains the best safeguard for patients with CLL.

## Author Contributions

**Enrica Antonia Martino, Santino Caserta, Fortunato Morabito, Massimo Gentile:** conceptualization. **Enrica Antonia Martino, Francesco Mendicino, Ernesto Vigna, Antonella Bruzzese, and Fortunato Morabito:** methodology. **Enrica Antonia Martino, Santino Caserta, Fortunato Morabito, Massimo Gentile:** writing – original draft preparation. **Enrica Antonia Martino, Santino Caserta, Fortunato Morabito, Massimo Gentile:** writing, review, and editing. All authors have read and agreed to the published version of the manuscript.

## Funding

The authors have nothing to report.

## Ethics Statement

The authors have nothing to report.

## Conflicts of Interest

The authors declare no conflicts of interest.

## Data Availability

Data sharing does not apply to this article, as no new data were generated or analyzed in this study.
